# West Chicago Medical Society

**Published:** 1881-04

**Authors:** 


### Article VIII.

West Chicago Medical Society. Stated meeting held March 14, 1881.

Dr. I. N. Danforth, President *pro tempore*, in the chair. An interesting paper was read by Dr. Mary H. Thompson. It related to the case of a Swedish woman whose *os uteri* had been deeply lacerated in a first confinement. This seemed to have been the result of an unskillful use of the forceps, and it occasioned dysmenorrhœa afterward. After a third confinement, in the course of which the *os* had to be incised and the forceps used, she began to have convulsions, for which Dr. Thompson gave her bromide of potassium, and the tincture of chloride of iron. The condition of the mouth of the womb was observed, and the vaginal douche, together with the local application of the nitrate of silver, formed part of the treatment. But with the subsidence of the congestion of the neck, the body of the womb became retroverted. Convulsions returned after four months.

Dr. Thompson now operated for the lacerations, one of which extended up to the body of the womb, and it was not deemed prudent to carry the denudation to the bottom of the wound. It resulted in a partial union and a fistula. This was closed at a subsequent operation, and the uterus was also replaced. No convulsion returned.

Patient was confined November 26, 1880, at her tenth month. It was found that the neck of the womb was long and undilatable after a frequent recurrence of the labor pains. Dr. Thompson being called outside the city, Dr. Bartlett took charge of the case, and had Drs. Bogue and S. G. Clark in consultation. This proved a tedious labor. After the forces of the woman had begun to wane, they incised the *os*, introduced Simpson's forceps at the superior strait, and delivered the mother of a hydrocephalic child, who died soon afterward, but the woman made a good recovery. This case will be discussed at the next meeting.

### A CONFERENCE BETWEEN DRUGGISTS AND PHYSICIANS.

The Chicago druggists responded to an invitation of the society in a large number. Mr. S. L. Coffin, President of the College of Pharmacy, read the proposed Pharmacy bill, and it was opened for discussion. The druggists want to repeal the amendment of the Legislature, giving physicians the same advantages as druggists in the compounding of prescriptions. Some of them wanted an entire separation of the two professions, at least in the cities; but the bill would apply to all the State, and there was the difficulty.

Mr. G. P. Englehard proposed that the society unite with the druggists to repeal the above-mentioned amendment, and pass resolutions on that point.

Dr. R. S. Hall, Secretary, remarked that the druggists had come, by invitation, to inform the medical society on the provisions of the bill, but not to settle the relations between physicians and druggists.

The duplicating of prescriptions was discussed by Mr. George Buck, Dr. Lyman and Dr. Twining. It seemed from their conclusions that it had grown into habit, and that a prescription is the property of a patient.

Dr. W. E. Clark, after stating the injustice of counter-prescribing, said that physicians had often to fall back on the old mode of furnishing remedies. That it was something very easy in our times, and that no medical college graduated a student in this country but was fully competent to compound medicines.

Dr. H. M. Lyman remarked that it would be very unjust to establish a law that would prevent the country physicians, and the Homœopathists as well, from dispensing their own remedies.

The most important point was reached when Dr. I. N. Danforth reproved the druggists for being all practitioners of medicine, and allowing their customers to call them doctors. Unless this was stopped the profession could not help them, and physicians would furnish their own remedies. Mr. A. G. Vogeler, R. H. Cowdrey and other druggists took part in the discussion. On the whole, the society thought the bill should be passed as it is, and amended afterward if that was proper.

The Secretary appointed Drs. Lyman, Clark and Twining a committee to report on such amendments after they had conferred with the Chicago College of Pharmacy. This report will be read at the next meeting, but the bill comes before the Legislature in a few days, and may be passed when the society meets the next time.

Dr. J. H. Tebbetts was elected a member. Through a mistake, his name was printed G. H. Peppitts in the March No. of the Journal.

---

Epilepsy and Crime.—In *Brain* quoted by London *Medical Record*, Dr. Clarke has published some very suggestive tables of statistics. He finds it hard to avoid the conclusion that alcoholism in the parents is a predisposing cause of crime and epilepsy. Forty-four per cent. of epileptic criminals were the children of drunken parents. With regard to the parents he finds that epilepsy is more frequent in the mother than in the father, and that the percentage for both parents is higher with the women than it is with the men. In drunkenness the reverse holds good. The proportion of epileptic and insane relatives is found to be very much greater with criminals than with ordinary epileptics. It has been asserted that "sexual desires show themselves early in the children of drunkards, and are associated with absence of moral sense." The author finds that the convictions for bastardy are three times as numerous among epileptics as among non-epileptics—a fact which strongly bears out his idea that epilepsy owes its origin to hereditary alcoholism. Other tables show that the amount of crime as indicated by the number of convictions is greater among epileptics than among ordinary criminals.—*Medical and Surgical Reporter.*

Dr. Edmund J. Doering, late of the U. S. Marine Hospital service, and well known by his contributions to these pages to many of our readers, has resigned his position in the service. He has formed a copartnership with Dr. E. O. F. Roler, of this city, and expects next month to settle in Chicago. He will be welcomed by his old friends and will undoubtedly make many new ones.

